# Fibrocytes and the tissue niche in lung repair

**DOI:** 10.1186/1465-9921-12-76

**Published:** 2011-06-09

**Authors:** Annika Andersson-Sjöland, Kristian Nihlberg, Leif Eriksson, Leif Bjermer, Gunilla Westergren-Thorsson

**Affiliations:** 1 Lung Biology Unit, Dept. of Experimental Medical Science, Lund University, Sweden; 2 Lung Medicine and Allergology Division, Dept. of Clinical Medical Science, Lund University, Sweden

## Abstract

Human fibrocytes are bone marrow-derived mesenchymal progenitor cells that express a variety of markers related to leukocytes, hematopoietic stem cells and a diverse set of fibroblast phenotypes. Fibrocytes can be recruited from the circulation to the tissue where they further can differentiate and proliferate into various mesenchymal cell types depending on the tissue niche. This local tissue niche is important because it modulates the fibrocytes and coordinates their role in tissue behaviour and repair. However, plasticity of a niche may be co-opted in chronic airway diseases such as asthma, idiopathic pulmonary fibrosis and obliterative bronchiolitis. This review will therefore focus on a possible role of fibrocytes in pathological tissue repair processes in those diseases.

## Introduction

Tissue repair and remodelling are ongoing processes in all types of wound healing. In healthy subjects, the primary role of the extracellular matrix (ECM) is to provide tissues with specific mechanical properties and to serve as a structural framework for cell attachment and migration. An ongoing tissue repair can result in fibrosis, which is regarded as an abnormal wound-healing process. Both resident tissue cells and recruited cells play significant roles in the pathological tissue repair.

Mesenchymal stem cells and progenitor cells have recently emerged as being important for maintaining tissue homeostasis. The dynamic relationship between the stem cells and the niche is very evident during tissue repair after an injury. Constitutive activation of repair programs, including accompanying inflammatory responses, leads to permanent changes in the niche that can lead to dysregulation of cellular function and stem cell behaviour. This can ultimately contribute to the disease progression, and therefore it is necessary to understand the molecular structure and composition of the niche to understand stem cell behaviour.

### Fibrocytes - markers, recruitment, and differentiation

A few years ago tissue-resident fibroblasts were thought to be the only possible source of fibroblasts. However, fibrocytes have recently been discovered as one of several different precursors of fibroblasts [1]. Epithelial-mesenchymal transition and endothelial-mesenchymal transition are also known to be possible sources of fibroblasts [2,3]. To evaluate the portion that each possible progenitor contributes to the fibroblast population, a bleomycin-induced model of lung fibrosis was studied. In this model, one-third of the fibroblasts were derived from epithelium and one-fifth from bone marrow. The proportions derived from endothelial-mesenchymal transition and from other possible origins were not investigated in this study [4]. Further studies are required to fully understand the mesenchymal origins of fibroblasts.

Fibrocytes are a distinct sub-population of bone marrow-derived fibroblast-like cells that can be found in the tissue and as circulating cells in peripheral blood. A combination of specific markers is used to identify fibrocytes such as combining haematopoietic markers with mesenchymal markers. For example, there are molecules specific for leukocytes (CD45), monocytes (CD11a, CD11b, CD13), and stem cells (CD34), and also chemokine receptors (CXCR4), major histocompatibility complex (MHC) molecules, and mesenchymal markers (prolyl 4-hydroxylase, α-smooth muscle actin (α-SMA)) on fibrocytes [1,5-8]. One of the most abundant markers is CXCR4, which is expressed by 90% of circulating fibrocytes [9]. The expression of these specific proteins alters as the fibrocytes are released from the bone marrow and recruited to the tissue. Mori *et al*. (10) isolated circulating fibrocytes from mice and analysed the cells regarding their CD13, CD34, CD45, collagen I, and α-SMA expression for one week in serum-free medium or in medium supplemented with transforming growth factor (TGF) -β, a factor involved in wound healing. The expression of CD13, CD34, and CD45 decreased, whereas the expression of collagen I was constantly high, and the expression of α-SMA was increased. The differences were even higher when TGF-β was present [10].

In the tissue, fibrocytes can also play a role in angiogenesis. For example, *in vitro*, fibrocytes produce a number of pro-angiogenic factors such as basic fibroblast growth factor (bFGF), vascular endothelial growth factor (VEGF), granulocyte-macrophage colony-stimulating factor (GM-CSF), interleukin (IL)-1, IL-8, and macrophage colony-stimulating factor (M-CSF). These factors induce migration, proliferation, and alignment of endothelial cells into tube-like structures [11]. Fibrocytes express matrix metalloproteinases (MMP)-2, MMP-7, MMP-8, and MMP-9, which can degrade ECM molecules. Such proteinases can also alter the behavior of intra- and extracellular proteins and further regulate the fibrocytes' possibility for proliferation, adhesion, migration, and chemotaxis [12].

**Figure 1:**
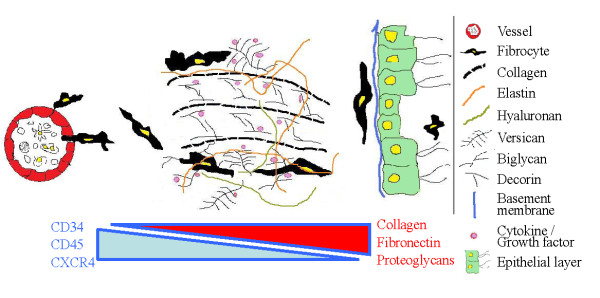
**Dynamic/temporal local micro-environmental niche**. The CXCR4-expressing fibrocytes are recruited from the circulation to the tissue by a gradient of stromal cell-derived factor (SDF)-1/CXCL12. In the tissue the fibrocytes move and interact with the dynamic/temporal local micro-environmental niche consisting of a broad array of extracellular matrix (ECM) molecules such as collagens, proteoglycans, hyaluronan, and glycoproteins. The ECM forms a network and acts as a supporting structure for tissue integrity. It is very essential that the ECM can also function as a reservoir for a large number of growth factors and cytokines, e.g., SDF-1/CXCL12, transforming growth factor (TGF)-β, interleukin (IL)-8, tumor necrosis factor (TNF)-α, platelet-derived growth factor (PDGF), and vascular endothelial growth factor (VEGF), just to mention some examples (generalized as violet spots in the figure) [55,56]. However, in disease states, the structure and composition of the ECM is changed, and that strongly affects the activity of cells in the niche. The alteration of the ECM, of course, is dependent on type of disease, state of disease, gene signature, age, gender, nutrition, infection, and also physical location in the pulmonary tree. It is important that the expression of the haematopoietic surface receptors, CD34, CD45, and CXCR4, on the fibrocytes gradually decreases, whereas the expression of the mesenchymal markers, such as collagen, fibronectin, and proteoglycans, gradually increases during the fibrocytes' journey through the tissue. The fibrocyte can also migrate to the lumen of the airway, and it has been detected there in both asthma and IPF.

Fibrocytes have to be recruited from the bone marrow to the injured tissue, and one of the possibilities for recruitment is the CXCR4-stromal cell-derived factor (SDF)-1/CXCL12 axis. SDF-1/CXCL12 belongs to the CXC family. The only receptor for SDF-1/CXCL12 is the G-protein-coupled seven-span transmembrane receptor CXCR4 [13], which is present on its target cell, e.g., the fibrocyte. Binding to receptor causes several changes to the fibrocyte: increased secretion of MMPs, VEGF, and nitric oxide (NO), as well as cytoskeletal rearrangements which give increased mobility and chemotaxis [14]. The surrounding ECM forms a micro-environment to which cells can attach, and the ECM forms the basement membrane located under the epithelial and endothelial cells. The main components of the ECM are collagens, proteoglycans, hyaluronan, and other glycoproteins. The ECM functions as a reservoir for growth factors and chemokines, and it is also a water-absorbent gel mass that gives the tissue its specific features. Under physiological conditions, the ECM turnover rate is highly controlled. Under pathological conditions, such as during tissue repair, there is a higher rate of synthesis and/or lower rate of degradation. The remodelling is closely associated with inflammatory processes, and some molecules involved in inflammation, such as hyaluronan, fibronectin, and fibrinogen, appear to increase the fibrocyte's sensitivity to SDF-1/CXCL12 [14,15] (Figure 1).

The importance of the CXCR4 - SDF-1/CXCL12 axis has been shown by Phillips *et al*. using a bleomycin model of lung fibrosis. Mice treated with anti-CXCL12 antibodies had significantly lower levels of collagen and α-SMA than mice treated with control antibodies [16]. Another possible mechanism for recruitment is a gradient of the chemokine secondary lymphoid tissue chemokine/chemokine ligand 21 (SLC/CCL21) [17]. It is normally expressed in lymphoid organs but is also found in lung tissue under inflammatory conditions. The receptor for SLC/CCL21 is CCR7, but it is only expressed by less than 10% of circulating fibrocytes [9]. This way of recruitment has been studied mostly in papers on renal fibrosis [5,18].

When fibrocytes have entered an injured tissue, they migrate through the tissue and are attracted to specific cytokines that are bound to the ECM. In disease-specific matrix (described below), the cytokine composition influences recruitment, differentiation and behaviour of fibrocytes, e.g., SDF-1 induces migration by interacting with CXCR4. The markers on the fibrocytes change during recruitment in the injured tissue. The expression of mesenchymal markers increases, while haematopoietic markers decrease [17] (Figure 1). In many ways fibrocytes differ from fibroblasts. An immunologically important difference is antigen presentation. Fibrocytes express both MHC class I and class II antigens and co-factors CD80 and CD86. Furthermore, fibrocytes can migrate to lymphatic organs and sensitise naive T-cells. Previously, this feature was only thought to be a task of dendritic cells [19].

Another possible goal for the differentiation of fibrocytes is to become adipocytes and chondrocytes. The differentiation to adipocytes is driven by specific adipogenetic hormones and cytokines which follow activation of specific adipocyte genes. On the other hand, TGF-β inhibits this differentiation by activating stress-activated protein kinase/c-Jun NH2-terminal kinase mitogen activated protein kinase (SAPK/JNK MAPK), which is normally suppressed during differentiation to adipocytes [20]. Furthermore, the differentiation to chondrocytes is driven by TGF-β3 together with a medium that differentiates mesenchymal stem cells to chondrocytes [21]. Interestingly, fibrocytes that have a chondrocyte-like phenotype show an increased expression of aggrecan and collagen [21].

### Tissue repair in lung disorders

**Figure 2:**
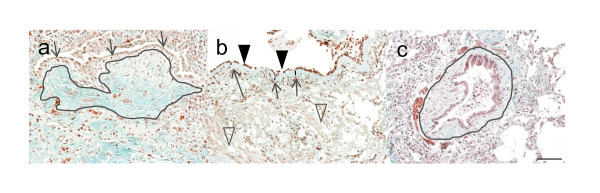
**Characteristic tissue niche in chronic airway diseases**. Panel (a) shows a histological section from a patient with IPF, characterized by a fibroblastic foci (outlined area) located below the epithelial layer (arrows). The fibroblastic foci is an area rich in ECM but with only few cells with a stretched morphology, located in parallel to the alveolar septa cells. Panel (b) shows a histological section from a patient with asthma, characterized by thickening of the basement membrane (arrows), shedding of the epithelial layer (closed arrowheads) and formation of peribronchial fibrosis (open arrowheads). Panel (c) shows a partially obliterated bronchiole (outlined area) in a patient with OB after lung transplantation. OB is histologically identified as ECM plugs with few fibroblasts. Original magnification 20×. Scale bar = 100 μm.

Today, both fibrocytes and fibroblasts are known to be important in wound healing as ECM-producing cells that function in response to injury. We also know that they can release cytokines and growth factors that are central for remodelling. In the lung, fibrosis can occur at different locations -- at the macro-level in the central part of the lung, at the micro-level in the distal alveolar parenchyma, and something in-between, in the small airways. In this review we have included three different patient groups, believed to differ somewhat in the primary site of fibrotic deposition. In asthma, the basement membrane, which is located below the epithelial layer, is thickened because of accumulation of collagens and proteoglycans [22] (Figure 2a). In idiopathic pulmonary fibrosis (IPF), fibroblastic foci occur in demarcated areas, which are rich in ECM and proteoglycans, but with few cells [23] (Figure 2b). In obliterative bronchiolitis (OB), the small airways are obliterated with ECM [24-26], where the proteoglycans function as "staples" to attach the connective tissue. In OB, the parenchymal part of the lung is also involved with thickening of the alveolar septa [27] (Figure 2c).

The above mentioned disorders are chronic diseases that involve remodelling of both the airways and the pulmonary vessels. The remodelling processes have many differences, but, surprisingly, also many similarities even though the underlying pathophysiological mechanisms are different. Remodelling usually starts with an epithelial injury that later gives rise to structural changes in the airways and in the lung. The origins of these disorders are different, but they have a common denominator -- the ECM deposition changes the lung structure, causes deterioration of the tissue, and thereby decreased lung function.

### Niche plasticity

#### Idiopathic Pulmonary Fibrosis

Many cell types are important in the pathology of IPF, but fibroblasts with their ability to produce matrix molecules are of special interest. Studies have shown that both the synthesis and the degradation of the ECM give rise to an ECM composition that is characteristic for fibrotic disease. In IPF, the tissue niche in the lungs contains approximately two to three times more ECM than healthy lung tissue, and the IPF fibrosis consists primarily of fibril-forming collagens (I, III, V, VI and VII), fibronectin, elastin, and proteoglycans [28] (Figure 1). Both proteases and inhibitors of those play an essential role in the degradation of the ECM. In tissues from patients with IPF, fibroblastic foci have been identified as discrete areas rich in ECM but with few cells. The cells in the fibroblast foci are arranged in an outstretched and parallel arrangement relative to the other cells and parallel to the alveolar septa [29] (Figure 2a). It has been speculated that fibroblast foci; play a key role in the destruction of the normal lung structure, are a negative prognostic factor and lead to the progressive and irreversible disorder [29]. Interestingly, the number of circulating fibrocytes is increased in patients with IPF, and the level is further elevated in patients during an acute exacerbation [30].

A possible origin of the fibroblasts in IPF is recruitment of fibrocytes from the bone marrow. The expression of CXCR4, and its ligand SDF-1/CXCL12, is known to be up-regulated under hypoxic conditions by hypoxia-induced factor 1α (HIF-1α) [31,32]. The bone marrow is hypoxic as compared to the surrounding vessels, and the bone marrow expresses SDF-1/CXCL12. An injury in the lung leads to increased levels of SDF-1/CXCL12 in the plasma in bronchoalveolar lavage fluid (BALF) [33], and fibrocytes are released from the bone marrow enabling them to migrate over a chemotactic gradient to the injured lung, where SDF-1/CXCL12 is expressed [34]. The fibrocytes express MMPs, which facilitate their transendothelial and tissue migration. Furthermore, the MMPs also act as a potential participant in the remodelling of the ECM [12].

The numbers of fibrocytes can be correlated to the structural changes in all three diseases. In IPF, the amounts of fibroblast/myofibroblast foci are a negative prognostic factor, the more foci, the worse the prognosis [35]. After normal wound healing, the fibroblasts and myofibroblasts should be reduced by apoptosis, but in IPF, and especially in fibroblast foci, the numbers of fibroblasts and myofibroblasts remain constant [36]. It has been speculated that fibroblastic foci, with their specific milieu, have a composition of cytokines, growth factors, and tissue inhibitor of metalloproteinases (TIMP) that cause fibroblasts and myofibroblasts to become apoptosis-resistant. Therefore, the ECM is produced in excess. However, the fibrocytes identified in the lung tissue of patients with IPF are not located inside the foci, but are located in close proximity to the foci in areas with ongoing signs of inflammation [33] Those adjacent areas would later become fibroblastic foci. One could speculate that fibrocytes that have been recruited towards the fibroblast foci already have been differentiated into fibroblasts or myofibroblasts during the migration.

#### Asthma

The remodelling and accumulation of ECM are also histological features of asthma, where many cell types with different features are involved. The structural cells involved in asthma include epithelial cells, smooth muscle cells, and (myo)fibroblasts. The fibrosis, and for asthma characteristic tissue niche, is subepithelial. There is a thickening of the lamina reticularis which contains collagens I, III, IV, VI, tenascin, and fibronectin [37-40]. This location is also abundant in fibroblasts and myofibroblasts in the asthma patient, while the proteoglycans, versican, biglycan, and decorin, accumulate in the submucosa below the epithelium in bronchial biopsies from asthma patients [38] (Figure 2b).

Nihlberg *et al*. have shown that there is both a central and a distal shift of the ECM composition, such as increased levels of versican and collagen, which the fibrocytes have to pass when they are recruited from the blood to the injured part of the lung. [22]. There, in the injured area, cytokines such as TGF-β, which are bound to the ECM, transform the fibrocytes to matrix-producing (myo)fibroblasts. Patients with chronic persistent obstructive asthma have higher levels of TGF-β and increased numbers of circulating fibrocytes than patients with asthma who have no loss of lung function [41].

A common technique to study fibrocytes *in vitro *is to cultivate them on fibronectin coated dishes, which allows the fibrocytes to attach to make further detailed analysis on biological activity and behaviour of these cells. Those type of studies have been performed of circulating fibrocytes from asthmatic patients, IPF patients, and healthy controls [9,20,42]. However, there are many questions to be solved concerning fibronectins' possibility to affect fibrocytes and the role of fibronectins in the tissue niche in these diseases [43].

Nihlberg *et al*. identified fibrocytes beneath the lamina reticularis in bronchial biopsies from patients with mild asthma, and the number of fibrocytes was correlated with the thickness of the basement membrane. The asthmatic patients were divided as to whether fibroblast-like cells could, or could not, be established from BALF. The patients with fibroblast-like cells from BALF showed both more fibrocytes in the tissue and increased numbers of eosinophils in BALF. It is possible that this is a result of on ongoing inflammation that contributes to fibrocyte recruitment [44].

#### Obliterative bronchiolitis

Obliterative bronchiolitis (OB) is a common consequence of both lung transplantation and bone marrow transplantation (affecting 60% and 6%, respectively) [45,46]. The tissue process starts with lymphocyte infiltration in the submucosa and injury of the mucosa and epithelial cell layer, and that results in recruitment of ECM-producing fibroblasts or their progenitor cells, such as fibrocytes. Histologically, the rejection is seen as an ECM plug with few fibroblasts in the bronchioles [24-26] (Figure 2c). The growth factors involved in the fibro-proliferative phase of the chronic rejection are platelet-derived growth factor (PDGF) [47] and TGF-β [48], which are known to up-regulate ECM deposition.

We found that at six months after lung transplantation the lung-tissue niche was changed. Versican and decorin production by fibroblasts was increased. After TGF-β stimulation, the fibroblasts produced even higher levels of versican and biglycan in patients that went on to develop OB as compared with patients without any signs of rejections [49].

There is a thickening of the alveolar parenchyma in patients with OB after lung or bone marrow transplantation. Furthermore, there is a correlation between the thickening and the greater number of fibrocytes in the tissue. Thickening of the parenchyma could give reduced lung function which is a criterion for OB. The vessels in OB patients are also remodelled in terms of increased amounts of endothelial layer and size of the lumen. There is a correlation between the remodelled vessels and the greater number of fibrocytes in the tissue [27].

### The common denominators of the remodelling are fibrocytes -- and, more speculatively, local hypoxia

The interactions between HIF-1α and HIF-1β, SDF-1 and CXCR4, VEGF and VEGFR during angiogenesis and hypoxia are known to be important in many diseases, including fibrotic disorders. The two subunits, HIF-1α and HIF-1β, together form a transcription factor that regulates expression of about 100 genes that are important in mechanisms such as anaerobic metabolism, angiogenesis, and apoptosis [50]. Under normal oxygen levels, HIF-1α is degraded and the complex with HIF-1β does not occur. Hypoxia increases the expression of SDF-1 in endothelial cells, epithelial cells, and in cells that are in stress after an injury. Furthermore, expression of its receptor, CXCR4 [51], is also elevated. A number of cells are known to express CXCR4 on their surfaces: fibrocytes, lymphocytes, muscle cells, and endothelial progenitor cells. Likewise, the expression of VEGF and its receptor, VEGFR, is also up-regulated to promote angiogenesis. The remodelled vessels, with enlarged lumen and greater endothelial cell area, that are identified in patients with OB after lung or bone marrow transplantation could in fact be a result of local hypoxia. Furthermore, an enlarged vessel gives a larger entrance area for the fibrocyte. The number of cells that co-express prolyl 4-hydroxylase and VEGFR2 is higher in patients with OB than in control individuals, and further, there is a correlation with the number of fibrocytes identified in the tissue (unpublished data). In asthma and IPF, vessel remodelling has also been studied, and in both diseases angiogenesis is involved that could be driven by hypoxic forces. In asthma, the vessels located in the bronchia and in the small airways are increased in number. In IPF the angiogenesis is dependent on an imbalance between IL-8 which is angiogenic, and IFN-γ, which is angiostatic [52-54].

### Fibrocytes in the lumen of the airway

After the fibrocytes have entered the tissue, the fibrocytes can differentiate into other cell types and/or continue to migrate to the lumen of the airway. Asthmatic patients and IPF patients differ regarding the types of cells found in the BALF. In asthmatic patients, a relatively high proportion of the fibroblast population expresses fibrocyte markers such as CD34, CD45RO, and α-SMA [44]. In the IPF patients, 1.0-3.4% of the cells were of mesenchymal origin. It is possible that this cell population is of fibrocytic origin but has differentiated because of the local environment in the IPF lung, and for this reason does not express CXCR4 (Figure 1). We are still missing data about fibrocytes in BALF from patients with OB. The pathophysiological obliteration of the small airways probably makes it difficult for fibrocytes to migrate to the lumen, at least in the occluded part of the lung.

## Conclusions

Each of the three diseases, asthma, IPF and OB, has its own specific local niche that influences the fibrocyte phenotype. In IPF, there is a correlation between the number of fibrocytes in the tissue and the number of fibroblastic foci. In asthma, thicker basement membranes are accompanied by fibrocytes in the BALF. In OB, there is a correlation between the number of fibrocytes and both vessel remodelling and thickening of the alveolar parenchyma. The fibrocytes can differentiate into fibroblasts which produce ECM molecules which further create or preserve each disorder's specific niche. Even thought we still do not know to what degree the fibrocytes contribute to each disease, it might be of interest to inhibit recruitment and differentiation of fibrocytes because they are associated with pathological airway remodelling.

## Competing interests

The authors declared that they have no competing interests.

## Authors' contributions

All authors wrote and revised the manuscript, and approved the final version.

## References

[B1] Bucala R Spiegel LA Chesney J Hogan M Cerami A Circulating fibrocytes define a new leukocyte subpopulation that mediates tissue repair Mol Med 1994 1 71 81 8790603 PMC2229929

[B2] Frid MG Kale VA Stenmark KR Mature vascular endothelium can give rise to smooth muscle cells via endothelial-mesenchymal transdifferentiation: in vitro analysis Circ Res 2002 90 1189 1196 10.1161/01.RES.0000021432.70309.28 12065322

[B3] Shook D Keller R Mechanisms, mechanics and function of epithelial-mesenchymal transitions in early development Mech Dev 2003 120 1351 1383 10.1016/j.mod.2003.06.005 14623443

[B4] Tanjore H Xu XC Polosukhin VV Degryse AL Li B Han W Contribution of Epithelial Derived Fibroblasts to Bleomycin Induced Lung Fibrosis Am J Respir Crit Care Med 2009 10.1164/rccm.200903-0322OC PMC2753790 19556518

[B5] Abe R Donnelly SC Peng T Bucala R Metz CN Peripheral blood fibrocytes: differentiation pathway and migration to wound sites J Immunol 2001 166 7556 7562 11390511 10.4049/jimmunol.166.12.7556

[B6] Aiba S Tagami H Inverse correlation between CD34 expression and proline-4-hydroxylase immunoreactivity on spindle cells noted in hypertrophic scars and keloids J Cutan Pathol 1997 24 65 69 10.1111/j.1600-0560.1997.tb01098.x 9162737

[B7] Quan TE Cowper S Wu SP Bockenstedt LK Bucala R Circulating fibrocytes: collagen-secreting cells of the peripheral blood Int J Biochem Cell Biol 2004 36 598 606 10.1016/j.biocel.2003.10.005 15010326

[B8] Strieter RM Gomperts BN Keane MP The role of CXC chemokines in pulmonary fibrosis J Clin Invest 2007 117 549 556 10.1172/JCI30562 17332882 PMC1804376

[B9] Mehrad B Burdick MD Strieter RM Fibrocyte CXCR4 regulation as a therapeutic target in pulmonary fibrosis Int J Biochem Cell Biol 2009 41 1708 1718 10.1016/j.biocel.2009.02.020 19433312 PMC2681415

[B10] Mori L Bellini A Stacey MA Schmidt M Mattoli S Fibrocytes contribute to the myofibroblast population in wounded skin and originate from the bone marrow Exp Cell Res 2005 304 81 90 10.1016/j.yexcr.2004.11.011 15707576

[B11] Hartlapp I Abe R Saeed RW Peng T Voelter W Bucala R Fibrocytes induce an angiogenic phenotype in cultured endothelial cells and promote angiogenesis in vivo FASEB J 2001 15 2215 2224 10.1096/fj.01-0049com 11641248

[B12] Garcia-de-Alba C Becerril C Ruiz V Gonzalez Y Reyes S Garcia-Alvarez J Expression of matrix metalloproteases by fibrocytes: possible role in migration and homing Am J Respir Crit Care Med 2010 182 1144 1152 10.1164/rccm.201001-0028OC 20622038

[B13] Horuk R Chemokine receptors Cytokine Growth Factor Rev 2001 12 313 335 10.1016/S1359-6101(01)00014-4 11544102

[B14] Kucia M Jankowski K Reca R Wysoczynski M Bandura L Allendorf DJ CXCR4-SDF-1 signalling, locomotion, chemotaxis and adhesion J Mol Histol 2004 35 233 245 15339043 10.1023/b:hijo.0000032355.66152.b8

[B15] Sbaa-Ketata E Courel MN Delpech B Vannier JP Hyaluronan-derived oligosaccharides enhance SDF-1-dependent chemotactic effect on peripheral blood hematopoietic CD34(+) cells Stem Cells 2002 20 585 587 10.1634/stemcells.20-6-585 12456967

[B16] Phillips RJ Burdick MD Hong K Lutz MA Murray LA Xue YY Circulating fibrocytes traffic to the lungs in response to CXCL12 and mediate fibrosis J Clin Invest 2004 114 438 446 15286810 10.1172/JCI20997 PMC484979

[B17] Lama VN Phan SH The extrapulmonary origin of fibroblasts: stem/progenitor cells and beyond Proc Am Thorac Soc 2006 3 373 376 10.1513/pats.200512-133TK 16738203 PMC2658688

[B18] Sakai N Wada T Yokoyama H Lipp M Ueha S Matsushima K Secondary lymphoid tissue chemokine (SLC/CCL21)/CCR7 signaling regulates fibrocytes in renal fibrosis Proc Natl Acad Sci USA 2006 103 14098 14103 10.1073/pnas.0511200103 16966615 PMC1599918

[B19] Chesney J Bacher M Bender A Bucala R The peripheral blood fibrocyte is a potent antigen-presenting cell capable of priming naive T cells in situ Proc Natl Acad Sci USA 1997 94 6307 6312 10.1073/pnas.94.12.6307 9177213 PMC21045

[B20] Hong KM Belperio JA Keane MP Burdick MD Strieter RM Differentiation of human circulating fibrocytes as mediated by transforming growth factor-beta and peroxisome proliferator-activated receptor gamma J Biol Chem 2007 282 22910 22920 10.1074/jbc.M703597200 17556364

[B21] Choi YH Burdick MD Strieter RM Human circulating fibrocytes have the capacity to differentiate osteoblasts and chondrocytes Int J Biochem Cell Biol 2010 42 662 671 10.1016/j.biocel.2009.12.011 20034590 PMC2835809

[B22] Nihlberg K Andersson-Sjoland A Tufvesson E Erjefalt JS Bjermer L Westergren-Thorsson G Altered matrix production in the distal airways of individuals with asthma Thorax 2010 65 670 676 10.1136/thx.2009.129320 20685740

[B23] Bensadoun ES Burke AK Hogg JC Roberts CR Proteoglycan deposition in pulmonary fibrosis Am J Respir Crit Care Med 1996 154 1819 1828 8970376 10.1164/ajrccm.154.6.8970376

[B24] Estenne M Maurer JR Boehler A Egan JJ Frost A Hertz M Bronchiolitis obliterans syndrome 2001: an update of the diagnostic criteria J Heart Lung Transplant 2002 21 297 310 10.1016/S1053-2498(02)00398-4 11897517

[B25] Nicod LP Mechanisms of airway obliteration after lung transplantation Proc Am Thorac Soc 2006 3 444 449 10.1513/pats.200601-007AW 16799090

[B26] Stewart S Fishbein MC Snell GI Berry GJ Boehler A Burke MM Revision of the 1996 working formulation for the standardization of nomenclature in the diagnosis of lung rejection J Heart Lung Transplant 2007 26 1229 1242 10.1016/j.healun.2007.10.017 18096473

[B27] Andersson-Sjoland A Erjefalt JS Bjermer L Eriksson L Westergren-Thorsson G Fibrocytes are associated with vascular and parenchymal remodelling in patients with obliterative bronchiolitis Respir Res 2009 10 103 10.1186/1465-9921-10-103 19878544 PMC2774308

[B28] Pardo A Selman M Kaminski N Approaching the degradome in idiopathic pulmonary fibrosis Int J Biochem Cell Biol 2008 40 1141 1155 10.1016/j.biocel.2007.11.020 18207447

[B29] Pardo A Selman M Idiopathic pulmonary fibrosis: new insights in its pathogenesis Int J Biochem Cell Biol 2002 34 1534 1538 10.1016/S1357-2725(02)00091-2 12379275

[B30] Moeller A Gilpin SE Ask K Cox G Cook D Gauldie J Circulating fibrocytes are an indicator of poor prognosis in idiopathic pulmonary fibrosis Am J Respir Crit Care Med 2009 179 588 594 10.1164/rccm.200810-1534OC 19151190

[B31] Ceradini DJ Kulkarni AR Callaghan MJ Tepper OM Bastidas N Kleinman ME Progenitor cell trafficking is regulated by hypoxic gradients through HIF-1 induction of SDF-1 Nat Med 2004 10 858 864 10.1038/nm1075 15235597

[B32] Phillips RJ Mestas J Gharaee-Kermani M Burdick MD Sica A Belperio JA Epidermal growth factor and hypoxia-induced expression of CXC chemokine receptor 4 on non-small cell lung cancer cells is regulated by the phosphatidylinositol 3-kinase/PTEN/AKT/mammalian target of rapamycin signaling pathway and activation of hypoxia inducible factor-1alpha J Biol Chem 2005 280 22473 22481 10.1074/jbc.M500963200 15802268

[B33] Andersson-Sjoland A de Alba CG Nihlberg K Becerril C Ramirez R Pardo A Fibrocytes are a potential source of lung fibroblasts in idiopathic pulmonary fibrosis Int J Biochem Cell Biol 2008 40 2129 2140 10.1016/j.biocel.2008.02.012 18374622

[B34] Christopher MJ Liu F Hilton MJ Long F Link DC Suppression of CXCL12 production by bone marrow osteoblasts is a common and critical pathway for cytokine-induced mobilization Blood 2009 114 1331 1339 10.1182/blood-2008-10-184754 19141863 PMC2727413

[B35] King TE Schwarz MI Brown K Tooze JA Colby TV Waldron JA Jr Idiopathic pulmonary fibrosis: relationship between histopathologic features and mortality Am J Respir Crit Care Med 2001 164 1025 1032 11587991 10.1164/ajrccm.164.6.2001056

[B36] Selman M Ruiz V Cabrera S Segura L Ramirez R Barrios R TIMP-1, -2, -3, and -4 in idiopathic pulmonary fibrosis. A prevailing nondegradative lung microenvironment? Am J Physiol Lung Cell Mol Physiol 2000 279 L562 L574 10956632 10.1152/ajplung.2000.279.3.L562

[B37] Davies DE Wicks J Powell RM Puddicombe SM Holgate ST Airway remodeling in asthma: new insights J Allergy Clin Immunol 2003 111 215 225 10.1067/mai.2003.128 12589337

[B38] Huang J Olivenstein R Taha R Hamid Q Ludwig M Enhanced proteoglycan deposition in the airway wall of atopic asthmatics Am J Respir Crit Care Med 1999 160 725 729 10430752 10.1164/ajrccm.160.2.9809040

[B39] Payne DN Rogers AV Adelroth E Bandi V Guntupalli KK Bush A Early thickening of the reticular basement membrane in children with difficult asthma Am J Respir Crit Care Med 2003 167 78 82 10.1164/rccm.200205-414OC 12502479

[B40] Malmstrom J Tufvesson E Lofdahl CG Hansson L Marko-Varga G Westergren-Thorsson G Activation of platelet-derived growth factor pathway in human asthmatic pulmonary-derived mesenchymal cells Electrophoresis 2003 24 276 285 10.1002/elps.200390024 12652599

[B41] Wang CH Huang CD Lin HC Lee KY Lin SM Liu CY Increased circulating fibrocytes in asthma with chronic airflow obstruction Am J Respir Crit Care Med 2008 178 583 591 10.1164/rccm.200710-1557OC 18583572

[B42] Saunders R Siddiqui S Kaur D Doe C Sutcliffe A Hollins F Fibrocyte localization to the airway smooth muscle is a feature of asthma J Allergy Clin Immunol 2009 123 376 384 10.1016/j.jaci.2008.10.048 19081612 PMC3992369

[B43] Hocking DC Fibronectin matrix deposition and cell contractility: implications for airway remodeling in asthma Chest 2002 122 275S 278S 10.1378/chest.122.6_suppl.275S 12475798

[B44] Nihlberg K Larsen K Hultgardh-Nilsson A Malmstrom A Bjermer L Westergren-Thorsson G Tissue fibrocytes in patients with mild asthma: a possible link to thickness of reticular basement membrane? Respir Res 2006 7 50 10.1186/1465-9921-7-50 16571120 PMC1458331

[B45] Belperio JA Weigt SS Fishbein MC Lynch JP III Chronic lung allograft rejection: mechanisms and therapy Proc Am Thorac Soc 2009 6 108 121 10.1513/pats.200807-073GO 19131536

[B46] Crawford SW Clark JG Bronchiolitis associated with bone marrow transplantation Clin Chest Med 1993 14 741 749 8313677

[B47] Hertz MI Henke CA Nakhleh RE Harmon KR Marinelli WA Fox JM Obliterative bronchiolitis after lung transplantation: a fibroproliferative disorder associated with platelet-derived growth factor Proc Natl Acad Sci USA 1992 89 10385 10389 10.1073/pnas.89.21.10385 1438225 PMC50343

[B48] El-Gamel A Sim E Hasleton P Hutchinson J Yonan N Egan J Transforming growth factor beta (TGF-beta) and obliterative bronchiolitis following pulmonary transplantation J Heart Lung Transplant 1999 18 828 837 10.1016/S1053-2498(99)00047-9 10528744

[B49] Andersson-Sjoland A Thiman L Nihlberg K Hallgren O Rolandsson S Skog I Fibroblast phenotypes and their activity are changed in the wound healing process after lung transplantation J Heart Lung Transplant 2011 in press 10.1016/j.healun.2011.04.006 21624839

[B50] Gardner LB Corn PG Hypoxic regulation of mRNA expression Cell Cycle 2008 7 1916 1924 10.4161/cc.7.13.6203 18604161

[B51] Ceradini DJ Gurtner GC Homing to hypoxia: HIF-1 as a mediator of progenitor cell recruitment to injured tissue Trends Cardiovasc Med 2005 15 57 63 10.1016/j.tcm.2005.02.002 15885571

[B52] Salvato G Quantitative and morphological analysis of the vascular bed in bronchial biopsy specimens from asthmatic and non-asthmatic subjects Thorax 2001 56 902 906 10.1136/thorax.56.12.902 11713351 PMC1745985

[B53] Keane MP Arenberg DA Lynch JP Whyte RI Iannettoni MD Burdick MD The CXC chemokines, IL-8 and IP-10, regulate angiogenic activity in idiopathic pulmonary fibrosis J Immunol 1997 159 1437 1443 9233641

[B54] Hashimoto M Tanaka H Abe S Quantitative analysis of bronchial wall vascularity in the medium and small airways of patients with asthma and COPD Chest 2005 127 965 972 10.1378/chest.127.3.965 15764783

[B55] Taylor KR Gallo RL Glycosaminoglycans and their proteoglycans: host-associated molecular patterns for initiation and modulation of inflammation FASEB J 2006 20 9 22 10.1096/fj.05-4682rev 16394262

[B56] Raghow R The role of extracellular matrix in postinflammatory wound healing and fibrosis FASEB J 1994 8 823 831 8070631 10.1096/fasebj.8.11.8070631

